# Artesunate versus quinine in the treatment of severe imported malaria: comparative analysis of adverse events focussing on delayed haemolysis

**DOI:** 10.1186/1475-2875-12-241

**Published:** 2013-07-15

**Authors:** Thierry Rolling, Dominic Wichmann, Stefan Schmiedel, Gerd D Burchard, Stefan Kluge, Jakob P Cramer

**Affiliations:** 1 Department of Internal Medicine, Section Tropical Medicine University Medical Centre Hamburg-Eppendorf, Hamburg, Germany; 2 Department of Clinical Research, Bernhard Nocht Institute for Tropical Medicine, Hamburg, Germany; 3 Department of Intensive Care Medicine, University Medical Centre Hamburg-Eppendorf, Hamburg, Germany

**Keywords:** Severe malaria, Imported malaria, Artesunate, Quinine, Haemolysis, Adverse events

## Abstract

**Background:**

Severe malaria is a potentially life-threatening infectious disease. It has been conclusively shown that artesunate compared to quinine is superior in antiparasitic efficacy and in lowering mortality showing a better short-term safety profile. Regarding longer-term effects, reports of delayed haemolysis after parenteral artesunate for severe malaria in returning travellers have been published recently. So far, delayed haemolysis has not been described after the use of parenteral quinine.

**Methods:**

In this retrospective study, all patients treated for severe malaria at the University Medical Centre Hamburg-Eppendorf were included between 2006 and 2012. The primary endpoint was the proportion of delayed haemolysis in patients treated with quinine versus those who received artesunate. As secondary endpoint, the proportion of any adverse event was assessed.

**Results:**

A total of 36 patients with severe malaria were included in the analysis. Of these, 16 patients contributed sufficient data to assess the endpoint delayed haemolysis. Twelve were treated primarily with intravenous quinine – with four patients having received intrarectal artesunate as an adjunct treatment – and five patients were treated primarily with artesunate. Five cases of delayed haemolysis could be detected – two in patients treated with quinine and intrarectal artesunate and three in patients treated with artesunate. No case of delayed haemolysis was detected in patients treated with quinine alone.

While adverse events observed in patients treated with artesunate were limited to delayed haemolysis (three patients, 60%) and temporary deterioration in renal function (three patients, 60%), patients treated with quinine showed a more diverse picture of side effects with 22 patients (71%) experiencing at least one adverse event. The most common adverse events after quinine were hearing disturbances (12 patients, 37%), hypoglycaemia (10 patients, 32%) and cardiotoxicity (three patients, 14%).

**Conclusions:**

This study provides further evidence on delayed haemolysis after artesunate and underlines the importance of a standardized follow-up of patients treated with artesunate for severe malaria.

## Background

Around 5,000 cases of imported *Plasmodium falciparum* malaria are notified per year in the WHO European region with around 25 deaths [1]. 562 cases (all plasmodial species combined) were reported to the Robert-Koch-Institute (RKI, Berlin, Germany) in 2011 with only one death (0.2%) [2]. *Plasmodium falciparum* malaria is a potentially life-threatening disease in Europe and prompt treatment is essential [3]. In endemic areas it has been shown conclusively that artesunate is superior in antiparasitic efficacy and in lowering mortality due to severe malaria when compared to quinine – showing beneficial effects especially in hyperparasitaemic patients [4-6]. In one retrospective study, superior efficacy of artesunate could also be confirmed in a European health care system setting [7]. In the updated German guidelines on diagnosis and treatment of malaria parenteral artesunate and quinine are now considered both as equivalent first-line treatment options [8]. Recently, artesunate has been advocated as the sole first-line treatment in any geographical region [3,6,9].

Regarding adverse events of parenteral artesunate, data is scarce so far [10]. This is in striking contrast to the existing safety evidence on quinine with its many known and well characterized adverse events such as cinchonism, hypoglycaemia or cardiotoxicity [11,12]. In the large multinational -QUAMAT trials, significantly less episodes of hypoglycaemia were observed after parenteral artesunate. However, while the rate of neurological sequelae was greater at discharge after artesunate, this difference waned over time and was attributed to the fact that a larger proportion of severely diseased patients with cerebral involvement survived after therapy with artesunate [5,6]. Recently, series of cases with delayed haemolysis in the second to third week after treatment with parenteral artesunate have been observed by several tropical medicine centres in hyperparasitaemic travellers returning to Europe [13-16]. This potential side-effect has not been reported in any of the large randomized trials comparing artesunate and quinine in endemic settings [4,5]. These trials were, however, not designed to detect any laboratory abnormalities in the follow-up period and the health care and social circumstances of malaria are not comparable to the situation in Europe [10].

Parenteral artesunate has received orphan drug designation for the treatment of malaria by the European Medicines Agency (EU/3/07/430 and EU/3/07/510). The orphan designation does not equate to a marketing authorization, but it offers incentives from the European Union to further develop and study a medicine for a rare disease such as malaria in Europe [17]. So far only artesunate produced by Guilin Pharmaceuticals (Shanghai, PR China) is available as WHO-confirmed Good Manufacturing Practice (GMP)-precertified drug. The French Medicines Agency has granted a temporary authorization of use with centralized data gathering in 2011 [18]. The lack of full GMP certification of artesunate, however, poses some liability issues in other countries such as Germany, where artesunate can only be administered on an individual treatment basis. In this scenario knowledge about potential adverse events of both treatment options as well as the evidence of antiparasitic superiority of artesunate are essential for patients and physicians to reach an informed decision.

The aim of this retrospective study was to assess adverse events in all patients treated for severe malaria since 2006 in the University Medical Centre Hamburg-Eppendorf with a special focus on the occurrence of delayed haemolysis.

## Methods

For this retrospective study, data were analysed from adult (≥18 years) patients, hospitalized between January 2006 and December 2012 at the University Medical Centre Hamburg-Eppendorf with a discharge diagnosis of severe malaria. Until 2011, quinine was recommended as the primary anti-malarial in severe malaria according to national guidelines while in 2011, artesunate was added as a first-line treatment option [8]. *Plasmodium falciparum* malaria was diagnosed by thin and thick blood film. Severe malaria was defined according to national guidelines and major severity criteria are shown in Table 1.

**Table 1:** Severity criteria according to the 2006 German guidelines for diagnosis and treatment of malaria

**Criterion**	**Definition**
Anaemia	Haemoglobin <8 g/dl
Acute renal failure	Urinary excretion <400 ml/24 h and/or creatinine >2,5 mg/dl or rapidly rising creatinine or Cystatin-C values
Hyperparasitaemia	>5% infected erythrocytes or >100.000 parasites/μl
Icterus	Bilirubin >3 mg/dl
Elevated liver function tests	>3x upper normal value
Impaired consciousness or convulsions	
Respiratory insufficiency or irregular respiration or hypoxia	
Hypoglycaemia	Blood glucose <40 mg/dl
Circulatory shock	
Spontaneous bleeding	
Acidosis	Base excess <-8 mmol/l
Hyperkalaemia	>5,5 mmol/l

Intravenous quinine (prepared by the pharmacy of the University Medical Centre Hamburg-Eppendorf) was given in combination with either oral doxycycline or oral clindamycin at a loading dose of 20 mg quinine dihydrochloride/kg (corresponding to 16,4 mg of quinine base) over four hours, followed by 10 mg quinine dihydrochloride/kg (corresponding to 8,2 mg of quinine base) every 8 hours until oral treatment was deemed possible with a total treatment period of seven days. Upon the discretion of the treating physician intrarectal artesunate (Plasmotrim®, Mepha Pharma, 5 doses of 200 mg) was added. Intravenous artesunate (Guilin Pharmaceuticals, Shanghai, China) was applied in four doses of 2,4 mg/kg (0 h, 12 h, 24 h, 48 h), followed by a full course of either oral mefloquine (3 doses every 8 hours of 750, 500 and 250 mg, respectively) or oral atovaquone/proguanil (1,000 mg/400 mg once daily for 3 days).

Primary endpoint was the proportion of delayed haemolysis in patients treated with quinine versus those who received artesunate. To detect delayed haemolysis and to separate this from haemolysis related to acute malaria, a case definition of a decrease in median haemoglobin (Hb) in combination with a rise in median lactate dehydrogenase (LDH) between week 2 (days 7 to 13) and week 3 (days 14 to 20) was chosen. Furthermore, the proportions of patients with additional side-effects likely associated with the anti-malarial medication were assessed.

Individual patient data were extracted retrospectively from paper charts until 2009. From 2010 onward individual patients’ data were retrieved from electronic hospital records. Patients’ records were screened for any disease- or treatment-related complications during the hospitalization and follow-up period. Laboratory parameters were entered into a database and analysed by SPSS 17.0 software package. Differences between groups were assessed by two-tailed Fisher’s exact test for categorical variables. Approval of the Ethics Committee of the Medical Council of Hamburg has been obtained.

## Results

A total of 44 patients fulfilling the criteria of severe *P. falciparum* malaria have been treated in the study period at the University Medical Centre Hamburg-Eppendorf. Of these, five patients (11%) treated with oral mefloquine and three patients (7%) treated with oral atovaquone/proguanil only were excluded from further analysis. Therefore, 36 patients who received either intravenous quinine or intravenous artesunate or both as primary parenteral anti-malarial were included. Table 2 shows all baseline demographic and clinical parameters. All patients acquired their plasmodial infection on the African continent. All but one patient (97%) returned from sub-Saharan Africa (25 from West Africa, five from Central Africa, three from Southern Africa and two from East Africa). Ghana (8) and Nigeria (6) were the most prevalent country destinations. One female Caucasian patient hospitalized for severe *P. falciparum* malaria in 2011 worked as a tourist guide and had travelled to several countries around the Mediterranean Sea including southern Europe, promed-note December 2010 (archive number: 2010121210.4401). Median length of hospital stay was 10 days (IQR: 7–18). Median baseline parasitaemia was 15,0% (IQR:7,0-25,0) and mean baseline Hb was 11,4 g/dl (95%CI: 10,2-12,6). Twenty-five patients (69%) were treated in an intensive care unit (ICU). One patient (3%) with an initial parasitaemia of 40% presenting with coma died. Despite rapid parasite clearance after treatment with intravenous quinine, intravenous doxycycline and intrarectal artesunate, he finally succumbed to multiple organ failure 36 days after initiation of anti-malarial treatment.

**Table 2:** Baseline demographic and clinical parameters

**Treatment group**	**All**	**Patients in whom delayed haemolysis could be assessed**			
	**All**	**All**	**Quinine (iv)**	**Quinine (iv) and artesunate (ir)**	**Artesunate (iv)**
n	36	16	8	4	4
Female	13 (36)	4 (25)	2 (25)	1 (25)	1 (25)
Visiting friends and relatives	10 (28)	5 (31)	3 (38)	1 (25)	1 (25)
Traveling from sub-Saharan Africa	35 (97)	16 (100)	8 (100)	4 (100)	4 (100)
ICU treatment	25 (69)	12 (75)	6 (75)	4 (100)	2 (50)
Deaths	1 (3)	0 (0)	0 (0)	1 (25)	0 (0)
Hospital stay duration in days, median (IQR)	10 (7–18)	17 (9–30)	19 (11–30)	17 (4–46)	11 (6–16)
Age in years, median (IQR)	49 (35–55)	48 (37–57)	42 (35–48)	55 (41–59)	54 (28–62)
Temperature in °C	38.1 (37.5-38.6)	38.1 (37.6 – 38.6)	38.2 (37.4- 39.0)	37.1 (37.0 – 37.2)	38.5 (37.6 – 39.4)
Parasitaemia in%, median (IQR)	15 (7–25)	15 (7–24)	12 (5–15)	33 (26–39)	17 (8,-21)
Hb (g/dl, normal range:12.0-16.0 )	11.4 (10.2-12.5)	11.4 (10.0 – 12.9)	11.9 (9.4-14.3)	9.8 (6.4-13.1)	12.3 (7.7-16.9)
WBC (1000/μl, normal range: 3.5 – 10.5)	6.9 (5.4-8.4)	8.1 (5.3-10.9)	7.8 (4.6-11.0)	11.6 (0.0-24.8)	5.5 (1.1-9.9)
Creatinine (mg/dl, normal range: 0.6 – 1.3)	1.6 (1.0-2.2)	2.0 (1.3-2.8)	2.0 (0.7-3.3)	1.7 (0.0-3.6)	2.5 (0.6-4.4)
Bilirubin (mg/dl, normal range: <1.2)	4.0 (2.7-5.2)	5.0 (3.9-6.1)	4.9 (3.0-6.8)	4.2 (0.0-10.3)	5.9 (3.3-8.5)
GOT (U/l, normal range: <50)	115 (71–158)	153 (102–205)	149 (46–251)	163 (21–305)	153 (55–251)
GPT (U/l, normal range: <50)	75 (52–98)	84 (57–111)	77 (40–115)	65 (8–120)	116 (4–228)
C-Reactive Protein (mg/l, normal range:<5)	182 (145–219)	178 (130–226)	187 (90–284)	178 (25–331)	163 (74–225)

Data are shown as number (%) or mean (with 95% standard deviation), if not indicated otherwise.

**Figure 1:**
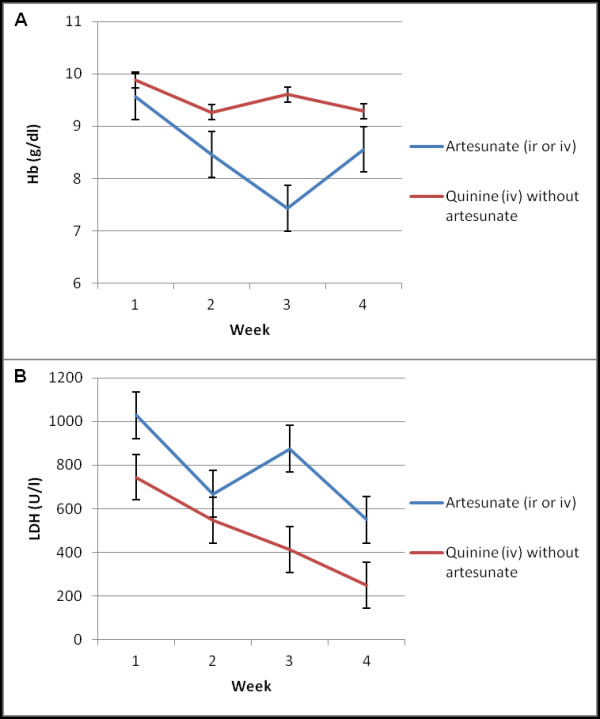
**Time course of median weekly Hb (A) and LDH (B) in patients treated with artesunate (n = 8) and in patients treated with quinine (n = 8).** ir: intrarectal; iv: intravenous. Mean ± standard error of the mean is displayed.

Table 3 summarizes the treatment regimens of patients included in this study. Thirty-one (86%) received intravenous quinine and five (14%) intravenous artesunate as their primary anti-malarial therapy. Data for Hb and LDH in weeks 2 and 3 were available for 16 patients (12 in the quinine group and four in the artesunate group) allowing the assessment of the primary endpoint definition of delayed haemolysis. In these 16 patients, five cases of delayed haemolysis were detected – two in patients treated primarily with quinine and three in patients treated primarily with artesunate. However, both patients developing delayed haemolysis after quinine had received additional intrarectal artesunate. Comparing the proportions of delayed haemolysis in patients who received intravenous artesunate with or without quinine (5/8, 63%) versus those who received intravenous quinine without artesunate (0/8, 0%), statistical significance was reached (p = 0,026). While delayed haemolysis was observed in patients treated with both, intravenous quinine and intrarectal artesunate, none of the patients treated with intravenous quinine without intrarectal artesunate showed the primary endpoint (2/4. 50%, versus 0/8, 0%; p = 0,091). Table 4 provides characteristics and clinical parameters of patients affected by delayed haemolysis. Three patients needed transfusions of packed red blood cells between days 14 and 21 (2 with primary quinine and 1 with primary artesunate treatment). Figure 1 illustrates the time course of Hb and LDH in patients having received any form of artesunate (intravenous or additionally intrarectal) and in patients having received quinine without any form of artesunate. Patients with delayed haemolysis reached their nadir in Hb around day 15 (range 15–17) in combination with a rise in haemolytic activity as shown by LDH peaks around day 15 (range 14–21). Direct Coombs test was performed in four of the five patients with delayed haemolysis. Two direct Coombs tests were negative; one showed a high titre of IgG warm-auto-antibodies and one a low titre of anti-E IgG antibodies. In the workup for haemolysis one patient was newly diagnosed with HIV. His CD4 count was 284 /μl with a viral load of 160,000 copies/μl.

**Table 3:** Treatment regimens of study patients

**Treatment regimen**	**All patients**	**Patients with delayed haemolysis,**
	***n*** **= 36**	***n*** = 5/16*
Quinine (iv) + Clindamycin (po)	9	0/4
Quinine (iv) + Doxycycline (po)	16	0/3
Quinine (iv) monotherapy	1	0/1
Quinine (iv) + Clindamycin (po) + Artesunate (ir)	1	1/1
Quinine (iv) + Doxycycline (po) + Artesunate (ir)	3	1/2
Quinine (iv) + Artesunate (ir)	1	0/1
Artesunate (iv) with subsequent Atovaquone/Proguanil (po)	4	2/3
Artesunate (iv) with subsequent Mefloquine (po)	1	1/1

*a total of *n* = 16 contributed data for the primary endpoint assessment (delayed haemolysis).

Abbreviation: *iv* intravenous, *po* orally, *ir* intrarectal.

**Table 4:** Demographic and clinical parameters of patients with post-treatment haemolysis

**Patient**	**Gender**	**Travel type**	**Country of infection**	**Anti-malarial treatment**	**Baseline parasitaemia**	**Other complications**	**Pre-existing medical conditions**	**Coombs test**	**Transfusions for post-treatment haemolysis**
1	f	Tourism	Burkina Faso	Quinine (iv) + Clindamycin (po) + Artesunate (ir)	30%	Acute renal failure (Peak creatinine: 8.5 mg/dl), QTc prolongation (550 ms)	Hypertension	DCT negative	Day 15 and Day 21 (2 units of packed red blood cells each)
2	m	VFR	Burkina Faso	Quinine (iv) + Doxycycline (po) + Artesunate (ir)	25%	Acute renal failure (Peak creatinine: 2.2 mg/dl)	Newly diagnosed HIV-1 infection	DCT positive, warm auto-antibodies (IgG)	Day 14 (2 units of packed red blood cells)
3*	f	Tourism	Uganda	Artesunate (iv) + Mefloquine (po)	14%	-	-	DCT not done	None
4*	m	Tourism	Gambia	Artesunate (iv) + Atovaquone/ Proguanil (po)	21%	Acute renal failure (peak creatinine: 6.5 mg/dl)	Hypertension	DCT positive, Low titre of anti-E IgG antibodies	Day 14 and Day 21, 2 units of packed red blood cells
5*	m	Tourism	Gambia/ Senegal	Artesunate (iv) + Atovaquone/ Proguanil (po)	20%	Acute renal failure (peak creatinine: 7.0 mg/dl)	-	DCT negative	None

*patients have been previously described in detail [15].

Abbreviation: *iv* intravenous, *po* orally, *ir* intrarectal.

Further adverse events are described in Table 5. The only complications recorded in patients treated with artesunate were delayed haemolysis and deterioration in renal function. In contrast, several complications were recorded in patients after quinine treatment: One or more adverse events were recorded in 22 (71%) quinine patients. Complications were manifold with 10 (32%) cases of hypoglycaemia requiring specific therapy, 12 (38%) cases of hearing disturbances and one case (3%) of visual impairment. In three patients with QTc-interval prolongation on ECG the quinine dose had to be reduced. Eight patients (26%) developed acute renal failure with four patients requiring haemodialysis. One patient (treated with doxycycline as combination drug) showed an increase in liver function tests (LFTs) during treatment and doxycycline had to be stopped – prompting a normalization of LFTs. One patient developed a syndrome of inappropriate antidiuretic hormone secretion (SIADH) and one patient developed an aseptic eosinophilic pneumonitis. These cases are described in more detail in Additional files 1 and 2.

**Table 5:** Reported adverse events other than delayed haemolysis in patients treated with a primary regimen of quinine and of patients treated with a primary regimen of artesunate

**Primary treatment**	**Quinine**	**Artesunate**
	***n*** **= 31**	***n*** **= 5**
Any adverse event	22 (71%)	3 (60%)
Hypoglycaemia (<50 mg/dl)	10 (32%)	0 (0%)
Hearing disturbances	12 (38%)	0 (0%)
Visual disturbances	1 (3%)	0 (0%)
Hepatotoxicity	1 (3%)*	0 (0%)
Prolongation of the QTc-interval (>500 ms)	3 (10%)	0 (0%)
Acute renal failure	8 (26%)	3 (60%)
Other**	2 (6%)	0 (0%)

* treated with quinine and doxycycline.

*** 1 patient with SIADH, 1 patient with clindamycin-induced eosinophilic pneumonitis.

All p-values for between-group differences >0,05 by Fisher’s exact test.

## Discussion

Delayed haemolysis was found only in hyperparasitaemic patients treated with artesunate (alone or in combination with quinine) but not in patients with quinine (and other partner drugs). After intravenous artesunate was established as a first-line treatment option for severe malaria in the University Medical Centre Hamburg-Eppendorf, active follow-up to detect haemolysis once a week for a total of four weeks after treatment initiation was implemented. The possibility that some cases of delayed haemolysis had been missed in quinine patients before establishing these active follow-up procedures cannot be ruled out. It appears realistic, however, to assume that delayed haemolysis would have been recognized passively in quinine patients if it was as frequent and clinically relevant. In addition, there are no other reports of delayed haemolysis after quinine available up to date. In contrast, increasing evidence indicates that delayed haemolysis occurs comparatively frequently after parenteral artesunate [13-16]. Interestingly we could detect delayed haemolysis also in two patients treated with intrarectal artesunate – a finding which had not previously been reported. It has to be noted that patients treated with quinine and additional intrarectal artesunate were more severely ill and had higher initial parasite densities than patients treated primarily with quinine. As hyperparasitaemia has been implicated as a risk factor for delayed haemolysis, parasite densities might be a confounder [14-16,19].

The aetiology of delayed haemolysis remains unknown. The fact that mainly hyperparasitaemic patients develop haemolysis may point to the contribution of a mechanism called ‘pitting’. After extraction of blood stage parasites during splenic passage, these once-infected erythrocytes have a reduced life-span compared to naïve erythrocytes with a mean life-span of around 180 hours and with a total removal of pitted erythrocytes after 28 days [20,21]. One could postulate an increase of haemolytic activity two weeks after acute malaria due to more or less synchronized destruction of pitted erythrocytes. The proportion of pitted erythrocytes seems to be higher after the use of artemisinins than after quinine – potentially explaining the absence of this adverse effect after use of quinine [20,21]. Immune-mediated haemolysis may be another explanation – although Coombs testing in the reports published so far has remained inconclusive. Coombs tests were negative in three patients in the case series by Zoller *et al.*[16]. In the Belgian-Dutch cohort, half of the tested patients had a positive Coombs test [14]. In the present study, half of the tested patients had positive direct Coombs test – albeit with different specificity (anti-E and warm autoantibodies). HIV-infection induced immunologic dysfunctions might have played a further role in the development of delayed haemolysis in one patient in this study. Further appropriately designed studies are necessary to clarify the pathophysiological mechanisms behind delayed haemolysis.

It is important to separate the haemolysis of acute malaria from delayed haemolysis after parasitological cure. Also, Hb levels can remain decreased during weeks two or three in patients slowly recovering from severe malarial anaemia without showing a new onset of haemolytic activity. It is, therefore, relevant to precisely define delayed haemolysis by applying biochemical parameters over time. In this study, delayed haemolysis was defined as a decrease in Hb (median Hb of week three lower than in week two) in combination with a rise in median LDH (median LDH of week three higher than in week two). This definition was selected based on the known time course of delayed haemolysis in the case series reported recently [13-16]. Median values of one week were used to correct for any potential outliers (e.g. transfusions or fluid resuscitation), which would have inappropriately influenced the mean values of one week.

The only other adverse event seen in patients treated with artesunate was development of acute renal failure after treatment was started. Renal failure might be due to malaria itself [22]. In an animal model, however, it was shown that intravenous artesunate leads to increased diuresis and decreased glomerular filtration rate [23]. Two case reports confirmed an increased diuresis in humans after infusion of intravenous artesunate – but no decrease in renal function was seen [24]. Creatinine levels in the three patients with acute renal failure after parenteral artesunate in this report normalized rapidly in the first week and no dialysis was necessary.

Quinine confers the risk of a wide spectrum of adverse events and complications. Cinchonism consisting of gastrointestinal disturbances, tinnitus, hearing loss, vasodilatation and headaches are comparatively common and reversible soon after the drug exposure has discontinued [12]. A potentially dangerous adverse event is hypoglycaemia – which occurred in every third patient in this study. Hypoglycaemic episodes are significantly more frequent in patients treated with quinine than in patients treated with artesunate [6]. Quinine (as well as quinidine – the prototypic drug of class Ia antiarrythmics) leads to changes in cardiac electrophysiology – mainly in a prolongation of the ventricular repolarization as emphasized by a prolongation of the corrected QT-interval and predisposing to the development of potentially lethal torsade de pointe arrhythmia [25]. Three patients developed prolongation of the QT-interval and the dose of quinine had to be reduced.

Several factors limit this study. Adverse events might be underreported in a retrospective chart review – either because patients do not or are unable to mention onset of new signs and symptoms because active screening is incomplete or simply because of incomplete chart entries. Furthermore, follow-up was not standardized for all patients which might hamper comparison between groups. Patients who were initially severely ill tended to have more extensive follow-up and adverse events might have been detected more frequently. These aspects may limit the generalizability of findings to patients with severe malaria. Moreover, the sample size was relatively small. A multi-centre retrospective approach might provide a larger sample size, although further heterogeneity in treatment regimens could add a further limitation. To define severe malaria, the German guidelines for the diagnosis and treatment of malaria issued in 2006 were used. In 2011, a new updated guideline has been published. In this revised guideline, elevated bilirubin and elevated LFTs have been omitted as severity criteria [8]. The 2006 guidelines were consistently applied for this study because it was the aim to yield a homogenous study population and because the majority of patients were treated prior to 2011.

## Conclusion

Delayed haemolysis seems to be a comparatively frequent as well as clinically relevant complication in severe malaria patients treated with parenteral artesunate. Yet, the fast and reliable antiparasitic action of artesunate outweighs the risks associated with delayed haemolysis including the necessity of blood transfusion, in particular in high standard medical care systems. While delayed haemolysis was not seen in patients treated with intravenous quinine (and partner drugs other than artesunate), quinine or respective partner drugs or both carry a significantly higher risk of adverse events and complications that may per se become life-threatening. Nevertheless, prolonged monitoring in particular for haematologic complications during at least one month after antiparasitic treatment initiation is strongly recommended after parenteral artesunate in patients with severe malaria. If this is warranted, parenteral artesunate should be used – if available – as the first-line treatment for severe malaria especially in hyperparasitaemic patients – despite lacking full GMP-conform production and licensure.

### Written informed consent

Consent was obtained from the patients for the publication of the two individual case reports.

## Competing interests

The authors declare that they have no competing interest.

## Authors’ contributions

TR and JPC designed the study, analyzed the data and wrote the first draft with contributions from all other authors. All authors were involved in the direct patient care of patients included in this study. All authors have read and approved the final manuscript.

## Supplementary Material

Additional file 1 **Case description – patient 1 **[26]**-**[29]**.** Click here for file

Additional file 2 **Case description – patient 2 **[30]**-**[33]**.** Click here for file
