# Supplementary material for: Artesunate versus quinine in the treatment of severe imported malaria: comparative analysis of adverse events focussing on delayed haemolysis
Source: Malar J. 2013 Jul 15;12:241. doi: 10.1186/1475-2875-12-241 (PMC3718719; doi:10.1186/1475-2875-12-241)
Supplement: Additional file 1 — Case description – patient 1 [26]-[29]. [file 1475-2875-12-241-S1.docx]

**Additional file 1**

One 63 old Ghanaian male with an initial parasitaemia of 15% treated successfully with quinine and clindamycin developed a severe delayed syndrome of inappropriate antidiuretic hormone secretion (SIADH). The patient noticed dysaesthaesias and movement disorders especially in the distal extremities around day 7 while serum sodium levels decreased. Minimal sodium level was 109 mmol/l on day 10, when the patient abruptly developed generalized seizures and was transferred to the intensive care unit. Sodium was initially substituted by hyperosmolar saline solutions and a fluid restriction regimen was started subsequently. Serum osmolarity was determined at 220 mmol/l with a urinary osmolarity of 608 mmol/l. Serum sodium was highly elevated with 129 meq/l (normal range: <40meq/l). Urea and creatinine were in the low normal range. No acid-base disturbances were noted. All these findings confirmed the diagnosis of SIADH.[26, 27] As sodium levels did not normalize with fluid restriction alone, treatment with tolvaptan (15mg once daily for 8 days) was started. Sodium levels normalized after stopping tolvaptan and neurological symptoms resolved.

Hyponatraemia is common in malaria - sometimes accompanied by an inappropriately high ADH level. However, in these cases hyponatraemia normalizes with declining parasitaemia in the majority of cases. [28, 29] In our case, however, SIADH developed only after parasite clearance. No alternative aetiology was found despite an extensive work-up. As SIADH is frequently associated with cerebral disorders, SIADH in this case might be considered a late neurological sequela after malaria. Interestingly, the patient recovered fully after a short course of the antidiuretic hormone receptor-antagonist tolvaptan.
