# Supplementary material for: Artesunate versus quinine in the treatment of severe imported malaria: comparative analysis of adverse events focussing on delayed haemolysis
Source: Malar J. 2013 Jul 15;12:241. doi: 10.1186/1475-2875-12-241 (PMC3718719; doi:10.1186/1475-2875-12-241)
Supplement: Additional file 2 — Case description – patient 2 [30]-[33]. [file 1475-2875-12-241-S2.docx]

**Additional file 2**

A 45-year-old French patient without any pre-existing medical conditions presented with cerebral malaria and an initial parasitaemia of 5% to a regional hospital in Northern Germany after returning from the Democratic Republic of Congo. Due to the inavailability of artesunate, treatment was started with intravenous quinine in combination with clindamycin and the patient was transferred to the Department of Tropical Medicine at the University Medical Centre Hamburg-Eppendorf. The treatment course with quinine and clindamycin was continued on the intensive care unit. Malaria was cured within one week. However the patient developed respiratory failure and required temporary mechanical ventilation one week after initiation of antiparasitic therapy. On computed tomography patchy ground-glass opacifications in both lungs were seen. As the patient did not ameliorate on empiric antimicrobial treatment with meropenem, bronchoalveolar lavage was performed. No pathogens could be cultivated, the cytology revealed marked pulmonary eosinophilia – compatible with the diagnosis of acute drug-induced eosinophilic pneumonia.[30, 31] After antimicrobial therapy with meropenem was stopped and a course of systemic corticosteroids (prednisolone 1mg/kg) was started, the respiratory situation recovered dramatically and the patient could be extubated. The course of disease was further complicated by spontaneous pneumothorax with subsequent formation of bullae requiring partial lung tissue resection. The patient recovered slowly during subsequent weeks until he was transferred back to Paris for continued hospital care.

A multitude of drugs – especially antibiotics – can cause pulmonary eosinophilia. Drug-induced pulmonary eosinophilia can vary in its severity from asymptomatic eosinophilia to respiratory failure – as in this case. [30, 31] Stopping the offending drug and starting a course of corticosteroids leads to a dramatic improvement in most cases and relapses are rare[30]. Clindamycin has been implicated in the development of eosinophilic pneumonitis in the past and in the drug review seems the most likely causative drug in this patient [32, 33]. This case shows that besides the primary treatment with artesunate or quinine the side effect profile of the partner drug has to be considered as well.
